# Mother-to-Child Transmission of HIV Infection and Its Determinants among Exposed Infants on Care and Follow-Up in Dire Dawa City, Eastern Ethiopia

**DOI:** 10.1155/2016/3262746

**Published:** 2016-02-16

**Authors:** Fisseha Wudineh, Bereket Damtew

**Affiliations:** ^1^Pediatric ART Clinic, ART Department, Dil Chora Referral Hospital, Dire Dawa, Ethiopia; ^2^Department of Epidemiology and Biostatistics, College of Health and Medical Sciences, Haramaya University, Harar, Ethiopia

## Abstract

Since the scale-up for prevention of mother-to-child transmission (PMTCT) services, rates of HIV infection among exposed infants have significantly declined. However, current achievements fell short of achieving the target sets. We investigated mother-to-child transmission (MTCT) of HIV infection and its determinants among HIV-exposed infants on care at Dilchora Referral Hospital in Dire Dawa City Administration. A retrospective institutional cohort study was conducted by reviewing follow-up records of HIV-exposed infants who were enrolled into care. Infants' HIV serostatus was the outcome measure of the study. Bivariate and multivariate logistic regressions were employed to identify significant determinants. Of the 382 HIV-exposed infants enrolled into care, 60 (15.7%) became HIV positive. Rural residence (AOR: 3.29; 95% CI: 1.40, 7.22), home delivery (AOR: 3.35; 95% CI: 1.58, 8.38), infant not receiving ARV prophylaxis at birth (AOR: 5.83; 95% CI: 2.84, 11.94), mixed feeding practices (AOR: 42.21; 95% CI: 8.31, 214.38), and mother-child pairs neither receiving ARV (AOR: 4.42; 95% CI: 2.01, 9.82) were significant independent determinants of MTCT of HIV infection. Our findings suggest additional efforts to intensify scale-up of PMTCT services in rural setting and improve institutional delivery and postnatal care for HIV positive mothers and proper follow-up for HIV-exposed infants.

## 1. Introduction

In 2011, there were approximately 330,000 children newly infected with Human Immunodeficiency Virus (HIV); most of the infections being occurring in sub-Saharan African countries [1]. Ethiopia is among the ten countries in the world with the highest burden of HIV infections among children where mother-to-child transmission (MTCT) occurs in a third of infants born to HIV-infected mothers. In 2012, HIV prevalence at antenatal care clinics was 2.4% and MTCT ranging from 17% (at 6 weeks) to 30% (including breastfeeding), amounting to an estimated 13,000 new HIV infections among children [1, 2].

Since the Ethiopian Ministry of Health launched the national program for prevention of mother-to-child transmission (PMTCT) of HIV infection in 2001, the number of facilities with PMTCT service has reached 1,445 providing ARV prophylaxis for 10,302 HIV positive pregnant women and 4,945 exposed infants in 2011 [3]. Although prophylactic medication coverage during pregnancy has improved significantly in east and southern Africa, it is still lower than the 80% target due to limited healthcare provision during childbirth [1]. In Ethiopia, while only 40% of identified HIV positive pregnant women received ARV prophylaxis (24% of the estimated 42,900 in need), less than a quarter of newborns to HIV positive women received ARV prophylaxis and only 4,753 (11%) exposed infants received virological test for HIV within 6 weeks of birth [4]. Low coverage of PMTCT services has been a consistent problem as nearly 82% of women accessed ANC services at least once during their most recent pregnancy; PMTCT services were available only in 54% of all facilities. Furthermore, while 98% of pregnant women attending ANC clinics providing PMTCT services were counseled, a quarter were not tested for HIV, and even among those who were HIV positive, 60% were not provided with ARV prophylaxis for PMTCT [3, 4].

In resource-limited countries with high prevalence of MTCT of HIV infection, where PMTCT is not widespread and early infant diagnosis (EID) is still a challenge, assessing the risk factors for MTCT will help to decrease child morbidity and mortality and strengthen PMTCT programs [5]. With this regard, few studies have described the magnitude of MTCT and assess efforts made towards PMTCT [6–8]; there is dearth of evidence regarding factors determining MTCT HIV infection to infants born to HIV positive mothers. Therefore, this study determines prevalence of mother-to-child transmission of HIV infection and its determinants among exposed infants in care at Dire Dawa Referral Hospital.

## 2. Methods

### 2.1. Study Setting

This study was conducted at Dil Chora Referral Hospital, in Dire Dawa City Administration. Dire Dawa is located 515 Km to the east of the capital city, Addis Ababa. The city has one referral hospital; four private hospitals; fifteen (9 in the city and 6 in rural kebeles) health centers; four private higher clinics; and one specialized STI and HIV care clinic. The referral hospital provides comprehensive HIV treatment and care as well as PMTCT service. At the time of the study there were 419 infants at the HIV-exposed infants clinic.

### 2.2. Study Design

Retrospective institution based follow-up study design was used to investigate factors associated with HIV transmission among infants born to HIV positive mothers who were on follow-up at the HIV-exposed infant care clinic. A total of 382 HIV-exposed infants who received care and support between July 2005 and July 2013 at the exposed infant follow-up clinic were included in the study. The independent variables were maternal factors (such as on HAART or prophylaxis, place of delivery) and infant factors (such as feeding options, follow-up). The study's outcome measure was HIV serostatus at the end of the follow-up.

### 2.3. Participants

The source population was all infants on follow-up in the HIV-exposed infant care clinic at Dil Chora Referral Hospital. The study population was comprised of exposed infants who were on follow-up care between July 2005 and July 2013 at the clinic and for which a confirmatory HIV test was done. Exposed infants <6 weeks of age, lost to follow-up, transferred out, or dead without confirmatory test were excluded from the study. Minimum sample size determination was made by considering a vertical HIV transmission rate of 10% among exposed infants on care from a previous study [7]. Study participants were selected by simple random sampling using their unique National PMTCT identification number.

### 2.4. Data Collection and Quality Control

A structured data collection tool was developed to compile the required information by adopting the national HIV-exposed infant follow-up form. The data were collected by reviewing mothers' PMTCT and exposed infants' care follow-up records at the antenatal care (ANC) clinic and exposed infant follow-up clinics, respectively.

The data were collected by two experienced nurses (one from ANC and one from exposed infant clinic) trained on comprehensive HIV care, PMTCT, and exposed infant care and who are working at the respective clinics in Dil Chora Referral Hospital. The data collectors were trained on the data collection procedures for one day. A pretest of the data collection tool and data collection process was made at Sabiyan Health Center found in the city. The investigator oversaw the overall process. All completed data collection forms were examined for completeness, consistency, and clarity during data management, storage, and analysis.

### 2.5. Follow-Up and Measurements

According to the national HIV-exposed infant care guideline, HIV diagnosis of exposed infants is made by a positive virological test using Deoxyribonucleic Acid-Polymerase Chain Reaction (DNA-PCR) test at 6 weeks or as early as possible thereafter, or if she/he displays any severe classifications possibly due to HIV or has positive antibody test under 18 months and 2 or more of the following: oral thrush, severe pneumonia, or severe sepsis. The child will be referred to ART care clinic after positive HIV diagnosis [4]. ARV prophylaxis follow-up schedule of infants born to HIV positive mothers is at 6 hours after birth, at 6th day, and then at the 6th, 10th, and 14th week of life. Thereafter, it is on monthly basis until 6th month of age and every 3 months until age of 18 months for asymptomatic infants. Cotrimoxazole prophylaxis is usually started when the infant is aged 4–6 weeks [3, 4]. However, children were followed up for variable periods of time as their HIV infection serostatus may be declared at different times.

### 2.6. Data Analysis

Exploration of data was made to check for any inconsistencies, coding error, out of range, and missing values and appropriate corrections were made. Descriptive analyses of sociodemographic information, infant prophylaxis, and infant follow-up information, maternal PMTCT interventions, and infant final HIV serostatus were carried out. Bivariate logistic regression model was used to assess associations of independent variables with the outcome variable and calculate their crude odds ratios. Finally, all variables significant at *P* ≤ 0.2 in the bivariate model were included in the final multivariate logistic regression model and their adjusted odds ratios were calculated. Variables significant at *P* < 0.05 in the final model were considered as independent determinant factors for infant HIV serostatus. And crosstabs were done to show the rate of each variable with the outcome. All analyses were conducted using SPSS version 16.0 for Windows (SPSS® Inc., Chicago, IL, USA).

## 3. Results

A total of 382 HIV-exposed infants on care were included in the study. One hundred ninety-three (50.5%) were females and majority 341 (89.3%) were from urban areas. Regarding place of delivery, 344 (90.1%) of the HIV-exposed infants were delivered at a health institution.

Of the total infants born to HIV positive mothers, 298 (78.0%) took ARV prophylaxis at birth and 217 (56.8%) received Cotrimoxazole prophylactic treatment. During the follow-up, 276 (69.9%) infants were on exclusive breastfeeding while the 35 (9.2%) were on mixed feeding. From the infants on care, 40 (10.5%) of them had history of growth failure and two of them had red flag in the developmental staging.

At the start of exposed infant care, 379 (99.2%) mothers were alive and 283 (74.1%) were enrolled into care. Among the HIV positive mothers, 253 (66.2%) of them were receiving PMTCT intervention during pregnancy and at child birth, of which 100 (26.2%) were already on ART. Almost all, 378 (99.0%) mothers had normal breast condition during the follow-up (Table 1).

### 3.1. Rate of Maternal to Child HIV Transmission

During the follow-up, there was maternal to child HIV transmission in 60 (15.7%) of HIV tested infants; most of them 55 (91.7%) were confirmed by DNA-PCR. The transmission was higher in females than in males (17.6% versus 13.8%). Fifty percent of infants delivered at home were HIV positive compared to 11.9% among infants delivered at health institutions. The transmission rate was higher among infants who did not receive ARV prophylaxis at birth (45.2%) compared to those who received (7.4%) (Table 1).

### 3.2. Determinants of Maternal to Child HIV Transmission

In the bivariate logistic regression analysis, mother's place of residence, infant's place of delivery, infant ARV prophylaxis at birth, feeding practice, infant age at enrollment, maternal enrollment into care, and PMTCT mother-child pairs were all associated with mother-to-child HIV transmission.

In the multivariate logistic regression analysis, being rural resident (AOR: 3.29; 95% CI: 1.40, 7.22), delivery at home (AOR: 3.35; 95% CI: 1.58, 8.38), infant not receiving ARV prophylaxis at birth (AOR: 5.83; 95% CI: 2.84, 11.94), mixed feeding practice (AOR: 42.21; 95% CI: 8.31, 214.38), and mother-child pairs not on PMTCT were found to be the most important significant determinants of mother-to-child HIV transmission (Table 2).

## 4. Discussion

In this institutional retrospective study of exposed infants, 60 became HIV positive during the follow-up. The determinant factors of mother-to-child HIV transmission were rural residency, delivery at home, infant not receiving ARV prophylaxis at birth, and mixed feeding.

The prevalence rate HIV infection among exposed infants in this study was 15.7%, which was comparable to national prevalence rate of 17% [2] and to reports from similar studies in countries with resource-poor settings where the prevalence rate reported was 11%–21.8% [5, 9, 10]. In spite of unrelenting global efforts made to eliminate MTCT and create a generation born HIV-free, the prevalence rate HIV infection in developing countries is still higher owing to low availability of PMTCT services for HIV positive pregnant women, elective caesarean sections, and avoidance of breastfeeding due to economic and cultural problem [1].

In this study, infants born to HIV positive mothers from rural residence were three times at higher risk (AOR: 3.29; 95% CI: 1.40, 7.22) of acquiring HIV infection than those born to mothers from urban areas. This finding falls in between figures reported from similar studies [6, 7]. The observed risk difference could be attributable to limited number of ANC clinics providing PMTCT services in rural areas compared to urban ones coupled with better information and higher ANC attendance by pregnant mothers in urban areas [2]. Among mothers of HIV positive infants, 44% of mothers from rural areas had not received any ARV prophylaxis (ART or PMTCT) compared to 30% among mothers of urban residence.

Infants born at home had a threefold higher risk for HIV infection (AOR: 3.35; 95% CI: 1.58, 8.38) compared to those delivered at health institutions. This is comparable to other studies from developing countries [7, 9, 10]. This could be because the risk of MTCT of HIV infection is minimized when attending skilled delivery in health institutions as it avails opportunities to ARV prophylaxes to the mother during labor and to the newborn right after birth. Regarding home deliveries in this study, 74% of pregnant mothers received PMTCT and only 16% of newborns received ARV prophylaxis at birth.

ARV prophylaxis at birth was another determinant factor for MTCT of HIV infection. Infants who did not receive ARV prophylaxis immediately after birth were 5.8 times at higher risk of being infected with HIV than their counterparts (AOR: 5.83; 95% CI: 2.84, 11.94). This was similar to finding from other African countries [5].

Infants receiving mixed feeding were 42 times at higher risk of acquiring HIV infection compared to those receiving exclusive breastfeeding (AOR: 42.21; 95% CI: 8.31, 214.38). A number of similar studies from resource-limited countries have also reported mixed feeding as an independent predictor of HIV transmission [5, 7, 10, 11]. The possible justification for increased risk mixed fed infants would be that irritation of infant's immature gastrointestinal tract might facilitate entry of HIV viral particles within the mother's breast milk to the blood stream.

## 5. Limitations of the Study

This study has some limitations. Since the data was secondary, the information gathered was not complete for some infants or mothers. Not all maternal potential factors for vertical transmission were explored by the study. There were changes in PMTCT and HIV-exposed infant care protocols at different times but this research did not assess the difference with each protocol.

## 6. Conclusion

A higher prevalence of HIV infection was observed among exposed infants on follow-up at the HIV-exposed infant clinic. The risk of HIV transmission was higher among infants from rural areas, indicating scaling up of PMTCT program especially in rural settings. In addition, a higher risk of HIV infection among exposed infants delivered at home, infants who did not receive ARV after birth, and mixed fed infants suggests additional efforts by healthcare providers to intensify community mobilization and provide regular health education to HIV positive mothers, so as to increase PMTCT intervention, institutional delivery, and proper follow-up at the exposed infants care clinic. More prospective studies are needed to understand the influence of additional factors on MTCT, especially in rural settings.

## Figures and Tables

**Table 1:** Sociodemographic and clinical characteristics of HIV-exposed infants on follow-up care in Dire Dawa, Ethiopia, September 2014.

Variables	Total, *N* (%)	Negative, *N* (%)	Positive, *N* (%)
Sex of infant			
Male	189 (49.5%)	163 (86.2%)	26 (13.8%)
Female	193 (50.5%)	159 (82.4%)	34 (17.6%)
Infant age at enrollment			
≤6 weeks	319 (83.5%)	275 (86.2%)	44 (13.8%)
>6 weeks	63 (16.5%)	47 (74.6%)	16 (25.4%)
Residence			
Urban	341 (89.3%)	296 (86.8%)	45 (13.2%)
Rural	41 (10.7%)	26 (63.4%)	15 (36.6%)
Place of delivery			
Health institution	344 (90.1%)	303 (88.1%)	41 (11.9%)
Home	38 (9.9%)	19 (50.0%)	19 (50.0%)
PMTCT intervention			
On ART	100 (26.2%)	94 (94.0%)	6 (6.0%)
PMTCT	153 (40.0%)	133 (86.9%)	20 (13.1%)
None	129 (33.8%)	95 (73.6%)	34 (26.4%)
PMTCT regimen			
Already on HAART	100 (26.2%)	94 (94.0%)	6 (6.0%)
None	129 (33.8%)	95 (73.6%)	34 (26.4%)
sdNVP	91 (23.8%)	78 (85.7%)	13 (14.3%)
sdNVP + AZT + 3TC	55 (14.4%)	48 (87.3%)	7 (12.7%)
sdNVP + AZT for 4 weeks	7 (1.8%)	7 (100.0%)	0 (0.0%)
ARV prophylaxis at birth			
Yes	298 (78.0%)	276 (92.6%)	22 (7.4%)
No	84 (22.0%)	46 (54.8%)	38 (45.2%)
Infant ARV regimen			
None	84 (22.0%)	46 (54.8%)	38 (45.2%)
sdNVP	127 (33.2%)	114 (89.8%)	13 (10.2%)
sdNVP + AZT 4 wks	36 (9.4%)	34 (94.4%)	2 (5.6%)
AZT + sdNVP + 3TC	104 (27.2%)	98 (94.2%)	6 (5.8%)
Others	31 (8.1%)	30 (96.8%)	1 (3.2%)
Feeding practice			
ERF	80 (20.9%)	78 (97.5%)	2 (2.5%)
EBF	267 (69.9%)	233 (87.3%)	34 (12.7%)
MF	35 (9.2%)	11 (31.4%)	24 (68.6%)
Growth pattern			
Normal	342 (89.5%)	291 (85.1%)	51 (14.9%)
Growth failure	40 (10.5%)	31 (77.5%)	9 (22.5%)

^*∗*^AZT, zidovudine; sdNVP, single dose nevirapine; 3TC, lamivudine.

**Table 2:** Determinants of mother-to-child transmission of HIV infection among exposed infants on follow-up care in Dire Dawa, Ethiopia, September 2014.

Variables	COR (95% CI)	*P* value	AOR (95% CI)	*P* value
Residence				
Urban	1	—	1	—
Rural	3.80 (1.87, 7.71)	0.000	3.29 (1.40, 7.22)	0.012
Place of delivery				
Health institution	1	—	1	—
Home	7.39 (3.62, 15.10)	0.000	3.35 (1.58, 8.38)	0.010
ARV prophylaxis at birth				
Yes	1	—	1	—
No	10.36 (5.63, 19.09)	0.000	5.83 (2.84, 11.94)	0.000
Feeding practice				
ERF	1	—	1	—
EBF	5.69 (1.34, 24.24)	0.019	4.86 (1.11, 21.40)	0.036
MF	85.10 (17.62, 410.87)	0.000	42.21 (8.31, 214.38)	0.000
Infant age at enrollment				
≤6 weeks	1	—	1	—
>6 weeks	2.13 (1.11, 4.08)	0.023	0.48 (0.19, 1.26)	0.135
Mother enrolled				
Yes	1	—	1	—
No	3.13 (1.78, 5.49)	0.000	1.11 (0.35, 3.51)	0.814
PMTCT intervention				
On ART	1	—	1	—
PMTCT	2.36 (0.91, 6.09)	0.077	1.76 (0.60, 5.11)	0.282
None	5.61 (2.25, 13.98)	0.000	0.72 (0.16, 3.27)	0.667
PMTCT mother-child pairs				
Both on ARV	1	—	1	—
Either one on ARV	2.42 (1.10, 5.29)	0.027	1.60 (0.65, 3.93)	0.303
Neither on ARV	9.21 (4.69, 18.08)	0.000	4.42 (2.01, 9.82)	0.000
